# Genome sequences of three temperate actinobacteriophages from clusters FA and AS

**DOI:** 10.17912/micropub.biology.001444

**Published:** 2025-02-07

**Authors:** Jonathan G. Lawton, Alyssa D. Reddy, Victoria M. Herrera, Riley J. Strain, Elroie E. Mekonnen, Frank W. Stearns, Samuel G. Obae, Rivka L. Glaser

**Affiliations:** 1 Center for Vaccine Development and Global Health, University of Maryland School of Medicine, Baltimore, Maryland, USA; 2 Department of Biochemistry and Chemistry, Stevenson University, Owings Mills, Maryland, United States; 3 Honors Program, Stevenson University, Owings Mills, Maryland, United States; 4 Department of Biological Sciences, Stevenson University, Owings Mills, Maryland, United States

## Abstract

Three novel temperate siphoviruses, Juno112, KHumphrey, and ChuckDuck, were isolated from soil at Stevenson University using the bacterium *Arthrobacter globiformis* B-2979. Based on gene content similarity, Juno112 and KHumphrey are assigned to actinobacteriophage cluster AS3 and ChuckDuck to cluster FA. All three phages encode tyrosine recombinases, with ChuckDuck encoding two.

**Figure 1. Transmission electron micrographs of three Arthrobacter phages:**
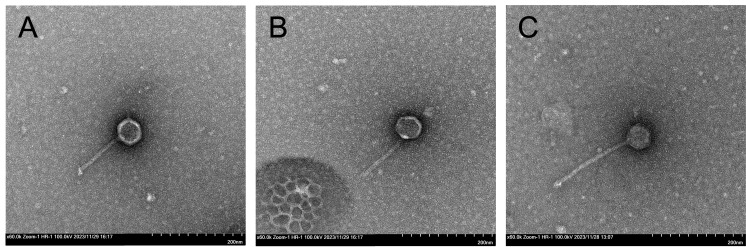
Juno112 (A), KHumphrey (B), and ChuckDuck (C) all have a siphovirus morphology, with long, non-contractile tails. The scale bar is 200 nm.

## Description

Discovering and characterizing novel bacteriophages advances our understanding of viral diversity and evolution and facilitates the development of molecular tools for manipulating bacteria. *Arthrobacter globiformis* is a soil-dwelling bacterium able to degrade various environmental pollutants (Megharaj et al., 2003, Amaresan et al., 2020) . Here, we describe three phages isolated using *A. globiformis* B-2979.

The three phages were isolated from soil samples collected at Stevenson University’s Owings Mills South campus in Owings Mills, Maryland, United States (Table 1) following an enriched isolation protocol (Zorawik et al., 2024) . Briefly, each soil sample was suspended in peptone-yeast extract-calcium (PYCa) liquid media, the suspension centrifuged (20 min x 4,000 g), the supernatant filtered (0.2 µm filter), and the filtrate inoculated with *A. globiformis* and incubated at 30°C for a 24-hour period. The resulting culture was refiltered and plated in top agar with *A. globiformis* to yield turbid plaques for Juno112 and clear plaques with well-defined borders for KHumphrey and ChuckDuck. Phages were purified through two rounds of plating before lysates were prepared. The phages were imaged using negative stain (1% uranyl acetate) transmission electron microscopy to reveal siphovirus morphology for all phages ( Fig. 1 ). Juno112, KHumphrey, and ChuckDuck have capsid diameters of 50 nm, 46.5 nm, and 54.5 nm and tail lengths of 106.3 nm, 116.3 nm, and 169.7 nm, respectively (n=1) ( Fig. 1 ).

DNA was extracted from phage lysates using the Promega DNA Wizard Cleanup Kit. Sequencing libraries were prepared using the NEB Ultra II FS DNA library kit and run on the Illumina MiSeq platform (v3 reagents), yielding 150 base single-end reads that were assembled into genomes using Newbler v2.9 and checked for completeness and genome termini using Consed v29 (Russell, 2018) . Sequencing data and genome characteristics are presented in Table 1.

Genome sequences of the three phages were annotated using DNA Master v5.23.6, build 270 (http://cobamide2.bio.pitt.edu/computer.htm). Protein coding sequences were detected using GeneMark v2.5p (Besemer and Borodovsky, 2005) and Glimmer v3.02b (Delcher et al., 2007) . Comparative genomic analyses against other phages in the PhagesDB actinobacteriophage database (Russell and Hatfull, 2017) were performed using Phamerator [Actino_Draft v564 database] (Cresawn et al., 2011) and Starterator v575 (phages.wustl.edu/starterator/). Gene function calls were determined using BLASTp searches against the PhagesDB and NCBI non-redundant protein databases (Altschul et al., 1990) , HHPred searches against the PDB_mmCIF70, SCOPe70, Pfam-A, NCBI_Conserved_Domains databases (Zimmerman et al., 2018) , Aragorn v1.2.38 (Laslett and Canback 2004) , and tRNAscanSE v2.0 (Lowe and Eddy, 1997) . Default settings were used for all software.

A total of 69 genes were predicted in Juno112, 38 of which were assigned putative functions, and KHumphrey had 70 predicted genes, 39 of which were assigned putative functions. ChuckDuck had 69 predicted genes, 30 of which were assigned putative functions (Table 1). Based on gene content similarity of more than 35% to phages in the Actinobacteriophage database, Juno112 and KHumphrey were assigned to phage cluster AS and subcluster AS3, whereas ChuckDuck was assigned to cluster FA (Pope et al., 2017) .

Characteristic of phages in subcluster AS3, Juno112 and KHumphrey contain 13 and 14 genes, respectively, in the center of the genome that are transcribed in the opposite direction from the remaining genes. This region includes immunity repressor and integrase genes, suggesting that these phages are able to establish lysogeny. ChuckDuck also encodes immunity repressor and integrase genes in the central region of the genome. The existence of lysogeny-associated genes is seemingly at odds with KHumphrey’s and ChuckDuck’s clear plaque formation, but this is a common discrepancy that is possibly explained by loss-of-function mutations disrupting the lysogenic cycle or key regulatory pathways (Gudlavalleti 2020) . Interestingly, two different tyrosine recombinases sharing only 25% amino acid identity are found in cluster FA phages, and ChuckDuck encodes both of these integrases, as well as a tRNA gene (Gly) and one gene for which no homologues exist to date.

The raw sequencing data for each phage are available in the Sequence Read Archive (Juno112: SRX25029055, KHumphrey: SRX25029056, ChuckDuck: SRX25029069). The assembled genomes are available in GenBank (Juno112: PQ362678, KHumphrey: PQ362674, ChuckDuck: PQ362676).

**Table 1: Isolation parameters, sequencing data, and genome characteristics**

**Table:** 

	**Juno112**	**KHumphrey**	**ChuckDuck**
**Soil Sample GPS Coordinates**	39.43N, 76.78W	39.42N, 76.77W	39.42N, 76.78W
**Plaque morphology**	Turbid	Clear	Clear
**No. of 150 base reads**	355,344	587,836	394,714
**Sequencing coverage, fold**	1394x	2347x	1457x
**Genome size (bp)**	38,469	38,343	42,833
**Genome termini**	3’single-stranded overhang 5’- GAGTTGCCGGCA	3’single-stranded overhang 5’- GAGTTGCCGGCA	3’single-stranded overhang 5’- CGCCGGAGA
**GC content (%)**	66.0	66.1	65.5
**No. of predicted genes**	69	70	69
**No. of genes with putative functions assigned**	38	39	30
**Cluster**	AS (subcluster AS3)	AS (subcluster AS3)	FA
